# Effect of niacin monotherapy on high density lipoprotein composition and function

**DOI:** 10.1186/s12944-020-01350-3

**Published:** 2020-08-21

**Authors:** Scott M. Gordon, Marcelo J. Amar, Kianoush Jeiran, Michael Stagliano, Emma Staller, Martin P. Playford, Nehal N. Mehta, Tomas Vaisar, Alan T. Remaley

**Affiliations:** 1 Saha Cardiovascular Research Center and Department of Physiology, University of Kentucky College of Medicine, 741 South Limestone, BBSRB Room B259, Lexington, KY 40536-0509 USA; 2 Translational Vascular Medicine Branch, National Heart, Lung, and Blood Institute, NIH, Bethesda, MD USA; 3 Section of Inflammation and Cardiometabolic Diseases, National Heart, Lung, and Blood Institute, Bethesda, MD USA; 4 Division of Metabolism, Endocrinology and Nutrition, Department of Medicine, University of Washington, Seattle, WA USA

**Keywords:** Niacin, Vitamin B3, High density lipoprotein, Apolipoproteins, Proteomics, Serum amyloid a, Cholesterol efflux

## Abstract

**Background:**

Niacin has modest but overall favorable effects on plasma lipids by increasing high density lipoprotein cholesterol (HDL-C) and lowering triglycerides. Clinical trials, however, evaluating niacin therapy for prevention of cardiovascular outcomes have returned mixed results. Recent evidence suggests that the HDL proteome may be a better indicator of HDL’s cardioprotective function than HDL-C. The objective of this study was to evaluate the effect of niacin monotherapy on HDL protein composition and function.

**Methods:**

A 20-week investigational study was performed with 11 participants receiving extended-release niacin (target dose = 2 g/day) for 16-weeks followed by a 4-week washout period. HDL was isolated from participants at weeks: 0, 16, and 20. The HDL proteome was analyzed at each time point by mass spectrometry and relative protein quantification was performed by label-free precursor ion intensity measurement.

**Results:**

In this cohort, niacin therapy had typical effects on routine clinical lipids (HDL-C + 16%, q < 0.01; LDL-C − 20%, q < 0.01; and triglyceride − 15%, q = 0.1). HDL proteomics revealed significant effects of niacin on 5 proteins: serum amyloid A (SAA), angiotensinogen (AGT), apolipoprotein A-II (APOA2), clusterin (CLUS), and apolipoprotein L1 (APOL1). SAA was the most prominently affected protein, increasing 3-fold in response to niacin (q = 0.008). Cholesterol efflux capacity was not significantly affected by niacin compared to baseline, however, stopping niacin resulted in a 9% increase in efflux (q < 0.05). Niacin did not impact HDL’s ability to influence endothelial function.

**Conclusion:**

Extended-release niacin therapy, in the absence of other lipid-modifying medications, can increase HDL-associated SAA, an acute phase protein associated with HDL dysfunction.

## Background

The cholesterol content of HDL (HDL-C) is an established biomarker for estimating risk of cardiovascular disease (CVD). This is based on the observations from the Framingham Heart Study and the subsequent “HDL hypothesis” suggesting that increasing HDL-C would provide protection against atherosclerosis [1, 2]. Over the last several decades, a variety of strategies have been evaluated for therapeutic elevation of HDL-C. Most recently, several trials evaluating cholesterol ester transfer protein (CETP) inhibitors have failed to reduce clinical events [3]. These results have called into question the relevance of HDL’s cholesterol content in the ability to protect against CVD and have helped the field to understand the distinction between HDL-C and HDL function.

In addition to cholesterol, HDL carry other lipid and protein cargo that likely play a more direct role in atheroprotection [4]. HDL carries about 100 different proteins with a wide range of known roles in inflammation, coagulation, and lipid transport [5, 6]. For the majority of these proteins, their impact on HDL function is not understood, but it has been suggested that these proteins can confer additional atheroprotective properties on HDL [7, 8]. It may be that the association of some of these proteins with HDL results in a paracrine-like effect, whereby HDL transports these proteins to specific tissues or cell types to achieve a physiological response. In this way, an increase in HDL size or particle number may also correlate with increased net protein transport and therefore a better paracrine-like effect, independent of cholesterol content. While HDL is generally thought of as protective, there is a growing body of literature that supports the existence of dysfunctional or pro-inflammatory HDL. These particles are generated under systemic inflammatory conditions and have impaired capacity for cholesterol efflux and reverse cholesterol transport, important atheroprotective functions of HDL that have been associated with reduced CVD events [9]. The impaired functionality of these particles is driven by replacement of the core HDL protein apolipoprotein A-I (apoA-I) with the acute phase protein serum amyloid A (SAA).

Niacin (vitamin B3) has a favorable impact on plasma lipid profile. Dosing at 1–2 g/day can have a modest effect on HDL-C, typically resulting in a 20% increase, while also reducing triglycerides and low-density lipoprotein cholesterol (LDL-C). Early evaluations of niacin monotherapy in reduction of cardiovascular disease appeared to be promising. In the 1960’s, the Coronary Drug Project, a randomized placebo-controlled secondary prevention study, demonstrated a benefit of niacin monotherapy with reduced cardiovascular mortality at 5 year and 15 year follow ups [10]. These findings were detectable despite a documented low adherence to niacin treatment. Modern forms of extended-release niacin and co-treatments, such as laropiprant, have been developed to reduce the flushing side effect of the treatment with the goal to improve adherence. More recent studies have examined the effect of extended-release niacin in combination with LDL-C lowering statins on cardiovascular outcomes in high-risk populations. Most notably, the AIM-HIGH (niacin + simvastatin) and HPS2-Thrive (niacin +laropiprant + statin) studies concluded that niacin did not offer additional benefit when added to standard statin therapy [11, 12]. The question of how niacin might differently affect HDL composition or function in the presence or absence of statins has not been answered.

The present study aimed to test the hypothesis that niacin has a direct effect on HDL composition and function by examining the effect of extended-release niacin monotherapy on HDL protein composition and function in a cohort of healthy volunteers who were not taking lipid modifying medications or supplements.

## Methods

### Subject recruitment and niacin administration

**Fig. 1:**
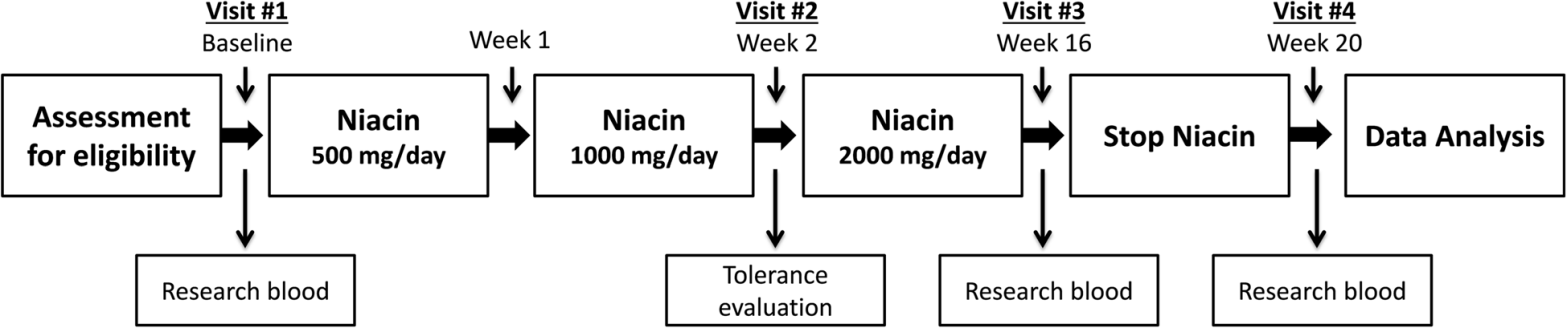
Study design. Schematic of the time course of the study. After qualifying for the study, eligible participants had a baseline visit followed by a two-week run in period where extended-release niacin was escalated to the target dose of 2000 mg/day. Tolerance was evaluated after week 2. Target dose was maintained until week 16 followed by a 4 week washout period. Research blood collected at baseline, week 16, and week 20 was used for lipoprotein proteome and functional assays

This pilot study took place at the National Institutes of Health Clinical Center. Male and female volunteers, who were not taking lipid modifying medications, were recruited to participate in this 20-week investigational study. Applicants were screened using the criteria listed in Table 1. After screening, enrolled volunteers received the dietary supplement Rugby® Extended Release Niacin (250 mg/tablet). An initial two week run-in period was used to slowly increase the dose from 500 mg/day up to the target 2000 mg/day (Fig. 1). The target dose was maintained for 14 weeks followed by a 4-week washout period.

**Table 1:** Subject Inclusion/Exclusion Criteria

**Inclusion Criteria** ● Males and females who are at least 18 years of age at time of enrollment, with fasting HDL-C below 60 mg/dL. ● Subject understands the investigational nature of the study and provides written, informed consent. **Exclusion Criteria** ● Subjects taking any lipid modification therapy, including but not limited to statins, fibrates and bile acid sequestrants. ● Subjects taking fish oil or any other supplements, which in the investigator’s opinion may interfere with the study. ● Subjects with acute liver disease or active peptic ulcer disease. ● Subjects with elevated uric acid levels or gout ● Pregnancy or women currently breastfeeding. ● Female subjects taking hormonal contraceptives or hormone replacement therapy may be included in this study only if they have been on a stable dose for at least 3 months. ● BMI less than 18.5 ● Subjects with weight that varies greater than 20% over the past 3 months BAS, antibiotics, anticoagulants, anticonvulsants, antiarrhythmic, Cyclosporine, Mycophenolate and Synthroid. Subjects with chronic diarrhea, gastric bypass or lap band procedures, ostomies, bowel motility problems, or other conditions that could affect intestinal fat absorption. ● Subjects initiating new medications or patients on multiple medications may also be excluded. ● Inability to swallow tablets ● Patients with a history of type I or type II diabetes or HbA1c > 6.5%. ● Volunteers may also be excluded, if in the opinion of the study investigators, they have some other condition or disorder that may adversely affect the outcome of the study or the safety of the volunteer.

The National Heart, Lung, and Blood Institute’s Institutional Review Board approved this study. All volunteers provided written informed consent for participation. ClinicalTrials.gov identifier: NCT02322203.

### Clinical lipid measurements

Blood was collected at baseline and during weeks: 2, 16 (niacin), and 20 (washout). Serum/Plasma was stored at -80 °C until analysis. Approximately 40 mL of fasting blood samples were collected at each visit and used to perform routine laboratory tests including a lipid panel, ApoA-I, and ApoB protein measurements and lipoprotein NMR profile. All tests were performed in the Department of Laboratory Medicine in the NIH Clinical Center (CC). Some of the collected blood was utilized for the in vitro cholesterol efflux assay and endothelial function test.

### HDL proteome analysis

Serum was thawed and filtered using 0.45 μm centrifugal filter units prior to purification of HDL on an Akta Pure FPLC system as previously described [5, 13]. Briefly, serum was separated over two Superdex 200 columns arranged in series at a flow rate of 0.5 mL/min. Fractions (0.5 mL/fraction) were collected and HDL-containing fractions pooled to produce total HDL for each subject. Lipid removal agent (LRA) was used to isolate HDL from lipid-free proteins. HDL proteins were subjected to trypsin digest while bound to LRA and peptides collected by washing. Peptides were desalted using ZipTips according to manufacturer's protocol, dried, and stored at − 20 °C. Dried peptide was reconstituted in 20 μL of water with 0.1% formic acid and 5 μL analyzed on an Orbitrap Fusion Lumos mass spectrometer (Thermo Scientific, Waltham (MA), USA). Sample was injected via a nanospray source over a 60 min. gradient from 5 to 70% acetonitrile followed by a 5 min. washing step at 90% acetonitrile. Solvent injection blank runs were performed between each sample to prevent sample carryover.

Label-free quantification of proteins was performed using MaxQuant software (version 1.6.0.16) [14, 15]. Raw files generated by the mass spectrometer were loaded into MaxQuant and searched against a subset database containing known HDL binding proteins using the incorporated Andromeda search engine. Settings included fixed carbamidomethyl modification and variable methionine oxidation. False discovery rates for peptide and protein were both set to 1%. These settings identified and produced quantification results for 71 proteins. This data was processed by exclusion of proteins which were not detected in at least 75% of subjects leaving 63 quantified proteins for comparison across treatment phases (Supplementary Table 1).

### Cholesterol efflux capacity

HDL efflux capacity was measured as previously described [13]. Assays were performed using the J774 murine macrophage cell line. Cells were plated (3 × 10^5^ cells/well) and loaded with 2 μCi of 3H-cholesterol/mL for twenty-four hours. ATP-binding cassette transporter A1 (ABCA1) expression was stimulated by sixteen-hour incubation with 0.3 mmol/L 8-(4-chlorophenylthio)-cAMP. ApoB-depleted plasma (2.8%) was added to the efflux medium and incubated for four hours. Efflux of radioactive cholesterol from the cells was quantified by liquid scintillation counting. Efflux % was calculated using the following formula: (μCi of 3H-cholesterol in media containing 2.8% apoB-depleted subject plasma-μCi of 3H-cholesterol in plasma-free media / μCi of 3H-cholesterol in media containing 2.8% apoB-depleted pooled control plasma-μCi of 3H-cholesterol in pooled control plasma-free media). The pooled control plasma was obtained from five healthy adult volunteers. All assays were performed in duplicate. Efflux data from two subjects was excluded from analysis due to severe outliers during measurement at one or more study phase time points. Outliers were detected by the ROUT method with a Q threshold of 2%.

### Endothelial function assay

Primary bovine aortic endothelial cells (BAEC) were used for the studies of HDL effect on endothelial function as described previously [16]. Cells were cultured in RPMI 1640 supplemented with 10% fetal bovine serum (Hyclone Laboratories, Logan, UT) and 12 μg/mL of bovine brain extract (Clonetics, Walkersville, MD), L-glutamine (2 mM), sodium pyruvate (1 mM) and nonessential amino acids in the presence of penicillin (100 units/mL) and maintained at 37 °C in 5% CO_2_. For the assays, the cells were plated in 24-well plates at 37 °C in 5% CO2. Prior to the assay, the cells were serum starved overnight (18 h) in media containing 0.1% FBS. The BAEC cells were then treated with 50 μg/mL HDL for 30 min and cell lysates were harvested in the presence of protease and phosphatase inhibitors. The cell lysates were run on 4–12% SDS-PAGE gels, transferred to pvdf membranes, and assayed by Western blot analysis for: phospho-Ser1179 eNOS (equivalent to human Ser1177) (Cell Signaling Technology, #9571), total eNOS (Thermo, 9DF10), Ser473 phos-Akt (Cell Signaling Technology, #4060) and total Akt (Cell Signaling Technology, #9272). The blots were scanned and the densitometry measurements from each gel were first normalized to a pooled normal HDL which was included in a random position on every gel to correct for gel-to-gel variability. Subsequently, for each sample, the signal of phosphorylated protein was normalized to total eNOS and Akt, respectively. To calculate % activation the corrected and normalized data was then normalized to the values obtained from control samples incubated with vehicle alone, and percent change relative to the control treatment condition was calculated.

### Statistical analysis

Statistical analyses were performed using GraphPad Prism software (version 7.05). For plasma lipid and lipoprotein measures, proteomics data, and HDL function assays, comparisons across study time points were performed by repeated measures one-way ANOVA with Geisser-Greenhouse correction and FDR correction by the method of Benjamini, Krieger, and Yekutieli. FDR adjusted q-values are reported. Values < 0.05 are considered statistically significant.

## Results

### Effect of niacin on plasma lipids and lipoprotein particles

Eleven volunteers completed this 20-week investigational study of extended-release niacin monotherapy (Fig. 1). Baseline characteristics are presented in Table 2. The cohort was 55% male with an average age of 40.4 years. At baseline, participants had normal blood pressure and were moderately overweight with a BMI of 28.8.

**Table 2:** Subject characteristics at baseline

n	11
Age (years)	40.4 ± 14.8
Sex (% male)	55
BMI (kg/m 2)	28.8 ± 6.6
Systolic BP (mmHg)	126.4 ± 11.5
Diastolic BP (mmHg)	72.0 ± 8.7
Total cholesterol (mg/dL)	220.1 ± 62.5
LDL cholesterol (mg/dL)	145.3 ± 63.3
HDL cholesterol (mg/dL)	52.1 ± 11.7

Values are mean ± standard deviation unless otherwise indicated

**Fig. 2:**
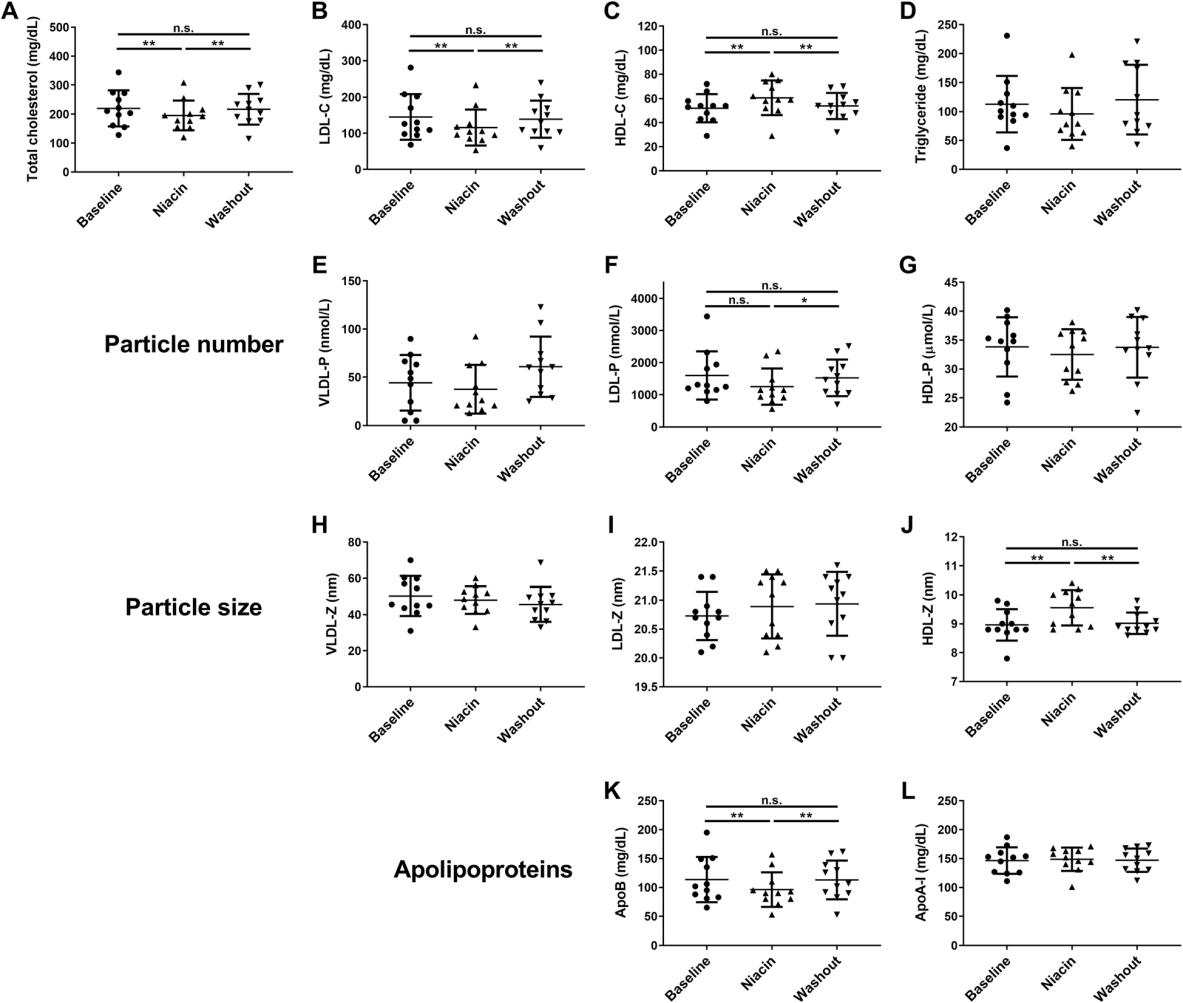
Effect of niacin therapy on plasma lipids, lipoprotein particles, and apolipoproteins. Lipoprotein profile was measured using nuclear magnetic resonance spectroscopy on a Vantera™ clinical analyzer (LabCorp). Plasma lipids (**a**-**d**), lipoprotein particle numbers (**e**-**g**), particle sizes (**h**-**j**), and apolipoprotein concentrations (**k**-**l**) were compared at baseline, on niacin (week 16), and washout (week 20). Comparisons were evaluated using repeated measures one-way ANOVA with false discovery rate (FDR) correction for multiple comparisons. * q < 0.05, ** q < 0.01, n.s. = not significant. If no indicators are present, then none of the comparisons was statistically significant

Plasma lipids were measured at baseline, on niacin (16 weeks on niacin), and after washout (4 weeks after stopping niacin). Total cholesterol was lowered by niacin (− 11%, q < 0.01) and returned to baseline during washout (Fig. 2a). The decrease in total cholesterol resulted from a 20% reduction in LDL-C (q < 0.01; Fig. 2b) and a 16% increase of HDL-C (q < 0.01; Fig. 2c). Both HDL and LDL returned to baseline following washout. Triglyceride levels were reduced by 15% (q = 0.1), although this was not statistically significant after multiple comparisons testing in this cohort (Fig. 2d). Lipoprotein profiling with NMR revealed details about particle number and size for each of the major lipoprotein classes. Consistent with the lack of effect on triglyceride, there was no change in VLDL particle number or size (Fig. 2 e,h). LDL particle number decreased by 22% (q = 0.05) on niacin although this was not statistically significant (Fig. 2f) and there was no effect on LDL particle size (Fig. 2i). Interestingly, HDL particle number did not change with niacin (Fig. 2g), however, HDL particle size increased by 7% (q < 0.01; Fig. 2j) likely reflecting the increased cholesterol content in the core of the particle. Plasma apolipoproteins reflected the changes observed with LDL and HDL particle numbers. ApoB was reduced on niacin and returned to baseline after washout (Fig. 2k), whereas apoA-I was not influenced by niacin (Fig. 2l).

### Niacin alters the HDL proteome

**Fig. 3:**
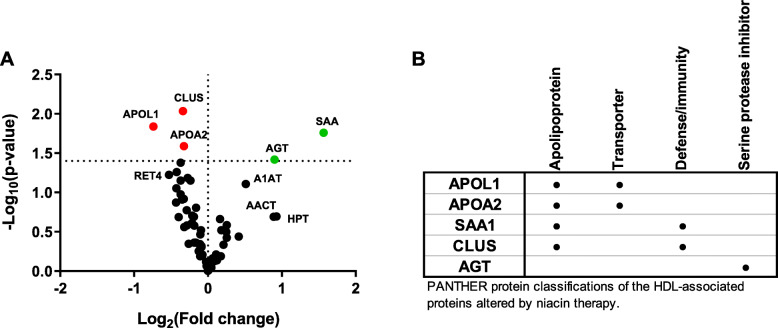
Niacin alters the HDL proteome. The HDL proteome was analyzed at baseline and after 16 weeks on niacin (2 g/day, extended release). (**a**) Changes to the HDL proteome are represented in a volcano plot. Each point indicates one of the 63 detected proteins. Proteins above the horizontal line were considered statistically significant changes. Green colored points indicate proteins increased while taking niacin and red points indicate reduced protein abundance while taking niacin. (**b**) Functional annotation of proteins affected by niacin was performed using Panther (version 14.1) gene list analysis [17, 18]

HDL was purified from plasma samples collected at baseline, niacin, and washout time points using a two-step approach designed to maintain physiological buffer conditions during isolation. Mass spectrometry was used to analyze the protein composition of HDL and a label-free quantitation strategy was used to compare protein abundance across samples. Compared to baseline, niacin administration for 16 weeks had a significant impact on 5 HDL-associated proteins (Fig. 3a). Three proteins were decreased including clusterin (CLU, commonly referred to as apoJ), apolipoprotein L1 (APOL1), and apolipoprotein A-II (APOA2). Two protein were positively associated with niacin administration, serum amyloid A (SAA) and angiotensinogen (AGT). Gene ontology analysis was used to provide general functional classifications to these proteins (Fig. 3b): apolipoprotein (4 of 5), transporter (2 of 5), defense/Immunity (2 of 5), and serine protease inhibitor (1 of 5).

### Effects of niacin on HDL protein composition are reversible

**Fig. 4:**
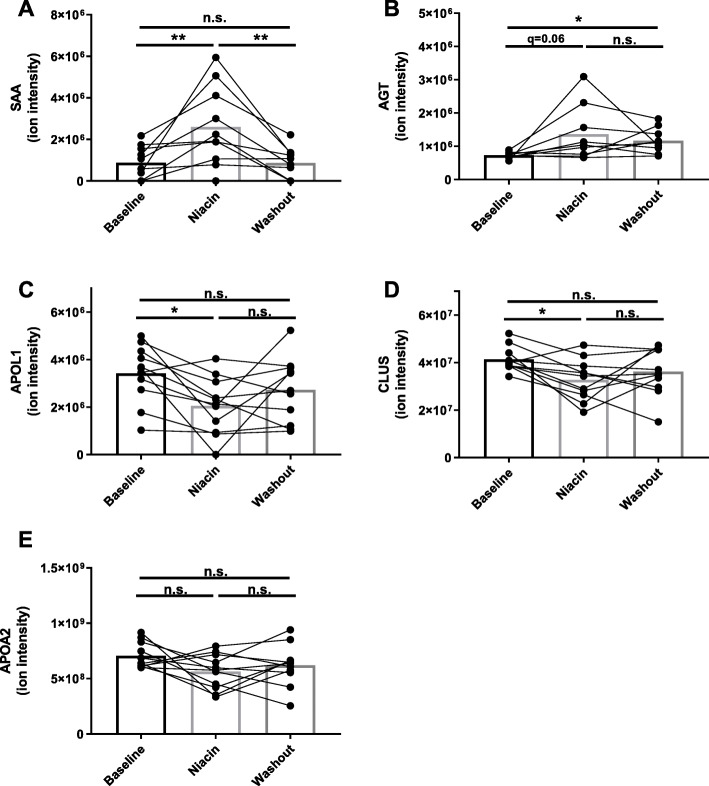
Effects of niacin on HDL protein composition are reversible. Relative protein abundance for the 5 proteins influenced by niacin were analyzed at baseline, on niacin (week 16), and washout (week 20). Changes in serum amyloid a (SAA; **a**), angiotensinogen (AGT; **b**), apolipoprotein L1 (APOL1; **c**), clusterin (CLUS; **d**), and apolipoprotein A-II (APOA2; **e**) were evaluated across time points using repeated measures one-way ANOVA with false discovery rate (FDR) correction for multiple comparisons. FDR adjusted probability value: * q < 0.05, ** q < 0.01, n.s. = not significant

The five proteins were analyzed by ANOVA to examine protein changes across the three treatment phases. The largest effect was seen with SAA, which increased 2.96-fold (q = 0.008) with niacin and completely returned to baseline during washout (Fig. 4a). AGT increased 1.87-fold (q = 0.06) and remained elevated (1.6 fold; q = 0.01) through the washout phase (Fig. 4b). APOL1 and CLUS both demonstrated significant reductions on niacin (− 40%, q = 0.01 and − 21%, q = 0.01, respectively) and partial return to baseline during washout (Fig. 4c,d). By this analysis, a 20% reduction in APOA2 on niacin was not statistically significant (q = 0.2) and its abundance trended back toward baseline levels during washout (Fig. 4e).

### Effect of niacin on HDL-mediated cholesterol efflux and endothelial function

**Fig. 5:**
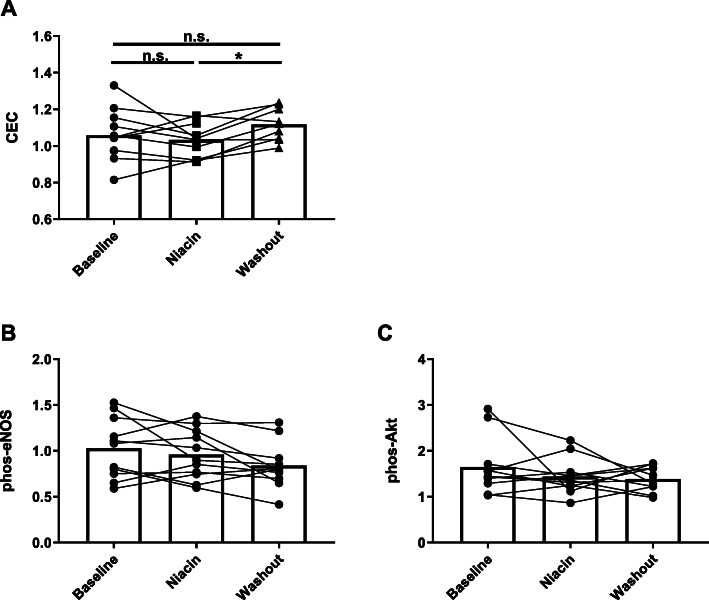
Niacin does not alter HDL-mediated cholesterol efflux or endothelial signaling. HDL function assays were performed on samples collected at baseline, on niacin (week 16), and washout (week 20). **a** Efflux of radiolabeled cholesterol from macrophage cells to apoB depleted serum. **b** Activation of endothelial cell signaling pathways eNOS and Akt was evaluated by measurement of phosphorylation after treatment with isolated HDL. Statistical comparisons were made using repeated measures one-way ANOVA with false discovery rate (FDR) correction for multiple comparisons. FDR adjusted probability value: * q < 0.05, n.s. or no indicator = not significant

To evaluate the impact of niacin montherapy on HDL function, cholesterol efflux capacity (CEC) and endothelial function assays were performed on samples collected from each time point. J774 macrophage cells were incubated overnight with cAMP to increase ABCA1 expression and loaded with radiolabeled cholesterol. Cholesterol efflux to apoB-depleted plasma was measured after a 4 h incubation. Niacin had no significant initial effect on cholesterol efflux, however, a rebound effect occurred after stopping niacin and a modest but significant increase in efflux (+ 9%, q = 0.01) was observed during the washout phase (Fig. 5a). For endothelial function assays, HDL was isolated from plasma by ultracentrifugation and incubated at 50 μg/mL with primary bovine aortic endothelial cells for 30 min. Cell lysates were analyzed by western blot for activating phosphorylation of eNOS (p-Ser1179) and Akt (p-Ser473). Phosphorylation of eNOS or Akt in endothelial cells was not affected by niacin (Fig. 5b,c).

## Discussion

This study tracked the influence of extended-release niacin on the protein composition of HDL in a small group of volunteers taking no additional lipid modifying medication. Plasma lipids in these participants displayed the typical response to niacin with decreased LDL-C (− 20%) and increased HDL-C (+ 16%), although the observed small reduction in triglyceride (− 15%) was not statistically significant. Label-free proteomics analysis detected niacin-induced changes in five HDL-associated proteins. Most notably, SAA was found to increase in response to niacin and return to baseline during the washout phase of the study. Despite previously reported effects of SAA on HDL function, we only detected a modest effect of niacin on cholesterol efflux capacity during the washout phase and no effect on endothelial functions of HDL in this study.

Using NMR lipoprotein profile analysis, it was determined that the effect of niacin on LDL-C lowering appears to be driven by a reduction in particle number without a change in average particle size. This is also supported by reduced plasma apolipoprotein B while taking niacin. Conversely, the niacin-induced increase of HDL-C is due to the generation of larger HDL particles and no change in total HDL particle number or plasma apoA-I was observed. Interestingly, these findings differ from patients receiving niacin + atorvastatin combination therapy where the same niacin dose (2 g/day) resulted in a 14% increase in HDL particle number. Although this can likely be explained by the much larger increase in HDL-C (39%) observed with combination therapy [19]. Although, in agreement with our study, combination therapy increased large and medium sized HDL particles and reduced small HDL particles, suggesting a net increase in average particle size. The effects of niacin on plasma lipids were reversible, returning to baseline levels after a 4-week washout period.

The change in HDL’s particle size was accompanied by changes to the proteome. SAA and AGT were increased on HDL in response to niacin. In humans, SAA is produced predominantly by the liver and has three isoforms. SAA1 and SAA2 are acute phase proteins whose plasma concentrations can increase by 200-fold during an inflammatory response. These isoforms are over 90% identical and because they are difficult to distinguish by usual mass spectrometry proteomics approaches, they are commonly grouped and referred to as SAA or SAA1/2. In general these isoforms are considered to function similarly and play important roles in response to injury and inflammation. SAA4 is constitutively expressed at relatively low levels and is not known to play a role in inflammatory response. The role of SAA on HDL has been investigated in detail. SAA can displace common apolipoproteins from HDL generating a dysfunctional particle with impaired cholesterol efflux and reverse cholesterol transport capacity [9, 20]. Although SAA was significantly elevated on HDL during niacin treatment, a significant reduction in efflux was not observed. This may be due to the relatively small increase of HDL-associated SAA induced by niacin monotherapy. Other conditions which significantly impair HDL cholesterol efflux by increasing HDL-associated SAA, such as endotoxemia, result in much higher levels of SAA on HDL [20]. However, during the washout phase of this study, efflux increased by 9% suggesting that there may have been a modest effect of niacin followed by a rebound effect. The effect of niacin + statin combination therapy on cholesterol efflux has been reported with mixed results. In one study, combination therapy resulted in increased total efflux, however, a modest reduction in ABCA1-specific efflux that did not reach statistical significance was observed [19]. Another study reported increased total cholesterol efflux in subjects treated with niacin + laropiprant + statin [21]. This study did not report ABCA1-specific efflux. Our study, using cAMP treated J774 macrophages, predominantly evaluates efflux mediated by ABCA1. In our study, it is not clear whether niacin treatment triggered a modest inflammatory response, which resulted in activation of the acute phase response and increased total plasma SAA or if niacin had a more direct effect on the HDL particle resulting in increased affinity for SAA. It was recently demonstrated that SAA is an exchangeable apolipoprotein, meaning that it can move among different lipoprotein classes [22]. Another possibility is that niacin treatment impacted the distribution of SAA among lipoprotein classes. The effect of extended-release niacin on endothelium-related functions of HDL has been examined in patients with type 2 diabetes [23]. HDL from these patients has reduced antioxidant and endothelial protective functions and these functions were largely improved with 3-month niacin treatment [23]. This study did not report the effect of niacin on these measures of HDL function in healthy individuals, so it is not clear if increases in these functions are possible in normally functioning HDL. While AGT may play an indirect role in HDL metabolism through regulation of scavenger receptor class B type 1 (SR-B1) expression [24], the importance of a physical association between AGT and HDL has not been investigated.

Three common apolipoproteins (CLUS, APOL1, and APOA2) were reduced in response to niacin. Among these, APOA2 (apoA-II) is the most abundant on HDL has been the most studied. ApoA-II is considered a core structural protein on HDL and particles have historically been segregated into species which contain apoA-I only or both apoA-I and apoA-II [25, 26]. Particles containing apoA-II have been suggested to carry more proteins with known roles in lipid transport while particles without apoA-II carry more proteins with roles in inflammation, immune response, and protease inhibition [27]. The physiological importance of interactions between CLUS or APOL1 with HDL have not been thoroughly investigated.

Many studies have now investigated the influence of disease condition or therapies on HDL composition and have detected alterations in the proteome [28]. However, it is still unclear what is physically causing these proteome shifts. One possibility is that the association or disassociation of a protein with HDL is driven by the local concentration of that protein in the plasma. In this case, the effect is indirect and not a result of direct action on the particle. Another possibility is that a direct change to the HDL particle has an impact on the association of one or more proteins. This could be a change in particle diameter that affects surface curvature or a change in surface lipid composition that influences the affinity of certain proteins. HDL remodeling is a complex process and a combination of direct and indirect effects may be occurring under different health conditions and even at different microenvironments within the circulatory system. Understanding the functional implications of HDL proteome remodeling is even more complex.

### Study strengths and limitations

One key strength of this study is the use of an unbiased label-free mass spectrometry approach to examine the HDL proteome in a well-defined cohort with high adherence to niacin treatment. The small sample size of this cohort is a limitation of the study, however, as a pilot study this cohort fulfilled our goal to detect key differences in the HDL proteome resulting from niacin. The inclusion of a washout phase was another strength that helped to provide confidence that detected differences were due to an effect of niacin. Because previous studies have examined the effect of niacin plus a stain on HDL composition and function it would have strengthened this study to have included a niacin plus statin arm for direct comparison of niacin monotherapy with niacin plus statin. This may be examined in future studies.

## Conclusion

In this small pilot study, niacin monotherapy caused proteome remodeling of HDL toward a particle enriched with SAA. SAA enriched HDL are described to be dysfunctional with regard to HDL’s atheroprotective properties and may even have proinflammatory activity. These findings suggest a potential mechanistic explanation for the mixed results of clinical trials of niacin in prevention of cardiovascular events. A larger study will be needed, however, to validate these findings in the general population and may uncover additional potential mechanisms to explain why the HDL-C increase induced by niacin does not appear to be cardioprotective. Evaluating other HDL-C raising strategies may reveal similar compositional effects on HDL function by generation of dysfunctional particles. Future attempts at clinical manipulation of HDL for CVD prevention should aim to improve the functional quality of HDL, rather than HDL-C. Although, which of HDL’s many functions is most important for future investigations is not clear. There is a clear disconnect between HDL-C and HDL function and despite a push to better catalogue the HDL proteome, a complete understanding of the complex dynamics between HDL proteome and HDL function does not exist.

## Supplementary information

**Additional file 1: Supplementary Table 1.** Label-free quantification of HDL-associated proteins.

## Data Availability

The datasets used and/or analysed during the current study are available from the corresponding author on reasonable request.

## References

[1] Gordon T, Castelli WP, Hjortland MC, Kannel WB, Dawber TR (1977). High density lipoprotein as a protective factor against coronary heart disease. Am J Med.

[2] Tariq SM, Sidhu MS, Toth PP, Boden WE (2014). HDL hypothesis: where do we stand now?. Curr Atheroscler Rep.

[3] Tall AR, Rader DJ (2018). Trials and tribulations of CETP inhibitors. Circ Res.

[4] Heinecke JW (2009). The HDL proteome: a marker--and perhaps mediator--of coronary artery disease. J Lipid Res.

[5] Gordon SM, Deng J, Lu JL, Davidson SW (2010). Proteomic characterization of human plasma high density lipoprotein fractionated by gel filtration chromatography. J Proteome Res.

[6] Vaisar T, Pennathur S, Green PS, Gharib SA, Hoofnagle AN, Cheung MC (2007). Shotgun proteomics implicates protease inhibition and complement activation in the antiinflammatory properties of HDL. J Clin Invest.

[7] Gordon SM, Hofmann S, Askew DS, Davidson WS (2011). High density lipoprotein: it's not just about lipid transport anymore. Trends Endocrinol Metab.

[8] Gordon SM, Remaley AT (2017). High density lipoproteins are modulators of protease activity: implications in inflammation, complement activation, and atherothrombosis. Atherosclerosis..

[9] Rosenson RS, Brewer HB, Ansell BJ, Barter P, Chapman MJ, Heinecke JW (2016). Dysfunctional HDL and atherosclerotic cardiovascular disease. Nat Rev Cardiol.

[10] Canner PL, Berge KG, Wenger NK, Stamler J, Friedman L, Prineas RJ (1986). Fifteen year mortality in coronary drug project patients: long-term benefit with niacin. J Am Coll Cardiol.

[11] Boden WE, Probstfield JL, Anderson T, Chaitman BR, Desvignes-Nickens P, Koprowicz K (2011). Niacin in patients with low HDL cholesterol levels receiving intensive statin therapy. N Engl J Med.

[12] Landray MJ, Haynes R, Hopewell JC, Parish S, Aung T, Tomson J (2014). Effects of extended-release niacin with laropiprant in high-risk patients. N Engl J Med.

[13] Gordon SM, Chung JH, Playford MP, Dey AK, Sviridov D, Seifuddin F (2018). High density lipoprotein proteome is associated with cardiovascular risk factors and atherosclerosis burden as evaluated by coronary CT angiography. Atherosclerosis..

[14] Cox J, Mann M (2008). MaxQuant enables high peptide identification rates, individualized p.p.b.-range mass accuracies and proteome-wide protein quantification. Nat Biotechnol.

[15] Tyanova S, Temu T, Cox J (2016). The MaxQuant computational platform for mass spectrometry-based shotgun proteomics. Nat Protoc.

[16] Vaisar T, Couzens E, Hwang A, Russell M, Barlow CE, DeFina LF (2018). Type 2 diabetes is associated with loss of HDL endothelium protective functions. PLoS One.

[17] Mi H, Muruganujan A, Huang X, Ebert D, Mills C, Guo X (2019). Protocol update for large-scale genome and gene function analysis with the PANTHER classification system (v.14.0). Nat Protoc.

[18] Mi H, Muruganujan A, Ebert D, Huang X, Thomas PD (2018). PANTHER version 14: more genomes, a new PANTHER GO-slim and improvements in enrichment analysis tools. Nucleic Acids Res.

[19] Ronsein GE, Hutchins PM, Isquith D, Vaisar T, Zhao X-QQ, Heinecke JW (2016). Niacin therapy increases high-density lipoprotein particles and Total cholesterol efflux capacity but not ABCA1-specific cholesterol efflux in statin-treated subjects. Arterioscler Thromb Vasc Biol.

[20] Vaisar T, Tang C, Babenko I, Hutchins P, Wimberger J, Suffredini AF (2015). Inflammatory remodeling of the HDL proteome impairs cholesterol efflux capacity. J Lipid Res.

[21] Yadav R, Liu Y, Kwok S, Hama S, France M, Eatough R (2015). Effect of extended-release niacin on high-density lipoprotein (HDL) functionality, lipoprotein metabolism, and mediators of vascular inflammation in statin-treated patients. J Am Heart Assoc.

[22] Wilson PG, Thompson JC, Shridas P, McNamara PJ, Beer MC, Beer FC (2018). Serum amyloid a is an exchangeable Apolipoprotein. Arterioscler Thromb Vasc Biol.

[23] Sorrentino SA, Besler C, Rohrer L, Meyer M, Heinrich K, Bahlmann FH (2009). Endothelial-vasoprotective effects of high-density lipoprotein are impaired in patients with type 2 diabetes mellitus but are improved after extended-release niacin therapy. Circulation..

[24] Wolf G, Wenzel U, Jablonski K, Brundert M, Rinninger F (2005). Angiotensin II down-regulates the SR-BI HDL receptor in proximal tubular cells. Nephrol Dial Transplant.

[25] Lund-Katz S, Murley YM, Yon E, Gillotte KL, Davidson WS (1996). Comparison of the structural and functional effects of monomeric and dimeric human apolipoprotein A-II in high density lipoprotein particles. Lipids..

[26] Silva RA, Schneeweis LA, Krishnan SC, Zhang X, Axelsen PH, Davidson WS (2007). The structure of apolipoprotein A-II in discoidal high density lipoproteins. J Biol Chem.

[27] Melchior JT, Street SE, Andraski AB, Furtado JD, Sacks FM, Shute RL (2017). Apolipoprotein A-II alters the proteome of human lipoproteins and enhances cholesterol efflux from ABCA1. J Lipid Res.

[28] Ronsein GE, Vaisar T (2019). Deepening our understanding of HDL proteome. Expert Rev Proteomic.

