# Supplementary material for: Effect of niacin monotherapy on high density lipoprotein composition and function
Source: Lipids Health Dis. 2020 Aug 21;19:190. doi: 10.1186/s12944-020-01350-3 (PMC7441610; doi:10.1186/s12944-020-01350-3)
Supplement: Supplementary file 1 [file 12944_2020_1350_MOESM1_ESM.docx]

Supplementary Table I. Label-free quantification of HDL-associated proteins.

	Baseline		Niacin		Washout	
**Protein**	**Average**	**Standard deviation**	**Average**	**Standard deviation**	**Average**	**Standard deviation**
**A1AT**	7272355	1742441	10366836	5242357	11981736	7925494
**A1BG**	5216455	1161186	5619809	2532338	6812955	3075066
**A2AP**	4409573	1645047	4041536	1227248	4458555	1081402
**A2MG**	1919018	714246	1916109	872098	1883809	499030
**AACT**	5324091	2562286	9864682	11182951	8696282	6200342
**AFAM**	1558782	372080	1694455	1226195	1989417	1013161
**ALBU**	4602672727	1329743999	4619772727	1940867335	5682254545	1795588808
**AMBP**	4307545	1721376	3446191	1883417	3712545	1712365
**AGT**	727588	93914	1358428	832878	1167407	377496
**ANT3**	24033000	10386053	28681545	13563099	31869000	13522063
**APOA1**	2423800000	582428756	2259448182	547327835	2114763636	648732693
**APOA2**	705330909	117571720	562687273	157286954	619308182	184531974
**APOA4**	189450909	52621278	150914909	58736279	147906364	36483018
**APOB**	1558536	427545	1601336	919042	1455307	459909
**APOC1**	8722555	3413689	6749873	1801976	6653800	1591865
**APOC2**	7035436	2828621	5215655	2633659	5849109	1800420
**APOC3**	37667182	12993222	33099136	13902572	30371909	10374658
**APOE**	17134636	4653915	13450136	6004046	14403082	5461409
**APOH**	46543364	10420788	50240182	21478535	43708091	15996883
**APOL1**	3420764	1208099	2051145	1191984	2722085	1340750
**APOM**	6408591	2513178	4767764	1711230	5401355	1656079
**C1S**	8658018	1715995	8147873	1723619	7804355	1683480
**CERU**	18884818	5621280	18912636	5023332	20006364	6961007
**CFAB**	163303636	38905359	143679091	41099812	155062364	45185969
**CFAH**	15608536	8919553	13019673	6680711	11572991	3607554
**CLUS**	41362000	5130518	32697273	8557648	36109091	9744129
**CO2**	4538809	493863	4061018	956995	4432555	1168249
**CO3**	751802727	110145522	637655455	164836851	667074545	102137101
**CO4A**	81359727	20576810	70240364	18464821	75549091	17455426
**CO9**	45186364	12243194	51215091	14350397	46677091	10249929
**FETUA**	78894455	18492536	74976909	18176966	79839818	18289990
**GELS**	47341545	5783327	44364000	7329297	44835636	9458904
**HEMO**	80118182	15411773	89733818	19769000	85797909	16204513
**HEP2**	28958000	5516555	28562000	4955866	26972364	4748134
**HPT**	72791000	44860971	138427227	158627305	140179836	197606096
**HPTR**	632635	368155	652668	428170	695929	407218
**HRG**	5439755	2412472	5054273	1327796	5231664	1534170
**IGHA1**	13580045	7574393	18130400	14402772	17479000	15177770
**IGHG1**	844591818	215586220	882376364	223602328	846104545	147651931
**IGHM**	1852808	946902	1407507	616642	1369944	564796
**IGKC**	50553455	13656026	60005909	27357920	51422818	18853561
**ITIH1**	20928455	5972501	16189791	5662915	17543545	4880307
**ITIH2**	62345364	16370077	46485000	19958628	56853364	23117980
**ITIH4**	17486545	7106055	14327182	1823463	15944091	3736541
**KAIN**	2375882	737727	2122482	752836	2533845	978529
**KLKB1**	15807091	3667391	16211091	2285219	15185636	3234663
**KNG1**	87959545	14112415	86355000	9931592	83259182	17378426
**LAC**	94787273	36771642	109635545	54731536	99011636	35620953
**LUM**	8733745	2253131	8107800	1522148	8546900	1880027
**PEDF**	5623791	1343529	5612500	1015553	5778755	1276156
**PGRP2**	3530827	1057689	3036855	865725	3034991	1357095
**PHLD**	3419927	907995	2996664	562419	2994109	777276
**PLMN**	55914909	9919302	52024273	8814875	50372909	9006741
**PON1**	29124727	4340753	27405818	6650766	27090091	4946219
**RET4**	5873882	2275250	4075818	1937228	5233085	1835792
**SAA**	878041	796384	2600534	1921669	867072	730176
**SAA4**	21879636	8704317	17946945	7264119	17826136	8534055
**SAMP**	230098	163868	259716	134224	313499	169666
**THRB**	12059964	2432467	9977764	2576644	10082873	2541535
**TRFE**	14496936	4472833	15456155	8541169	16763191	8517210
**TTHY**	8727118	3649131	8996000	3999032	10564100	3782836
**VTDB**	23877909	5999345	28473545	11697959	32988364	15823324
**VTNC**	41114818	9354087	31837309	10613366	34175636	10148815

Values are LFQ intensity calculated using MaxQuant (version 1.6.0.16).
